# Icaritin requires Phosphatidylinositol 3 kinase (PI3K)/Akt signaling to counteract skeletal muscle atrophy following mechanical unloading

**DOI:** 10.1038/srep20300

**Published:** 2016-02-02

**Authors:** Zong-Kang ZHANG, Jie LI, Jin LIU, Baosheng GUO, Albert LEUNG, Ge ZHANG, Bao-Ting ZHANG

**Affiliations:** 1 School of Chinese Medicine, The Chinese University of Hong Kong, Hong Kong SAR, China; Shenzhen Research Institute, The Chinese University of Hong Kong, Shenzhen, 518057, China; 2 Institute for Advancing Translational Medicine in Bone & Joint Diseases, School of Chinese Medicine, Hong Kong Baptist University, Hong Kong SAR, China

## Abstract

Counteracting muscle atrophy induced by mechanical unloading/inactivity is of great clinical need and challenge. A therapeutic agent that could counteract muscle atrophy following mechanical unloading in safety is desired. This study showed that natural product Icaritin (ICT) could increase the phosphorylation level of Phosphatidylinositol 3 kinase (PI3K) at p110 catalytic subunit and promote PI3K/Akt signaling markers in C2C12 cells. This study further showed that the high dose ICT treatment could significantly attenuate the decreases in the phosphorylation level of PI3K at p110 catalytic subunit and its downstream markers related to protein synthesis, and inhibit the increases in protein degradation markers at mRNA and protein levels in rat soleus muscle following 28-day hindlimb unloading. In addition, the decreases in soleus muscle mass, muscle fiber cross-sectional area, twitch force, specific force, contraction time and half relaxation time could be significantly attenuated by the high dose ICT treatment. The low dose ICT treatment could moderately attenuate the above changes induced by unloading. Wortmannin, a specific inhibitor of PI3K at p110 catalytic subunit, could abolish the above effects of ICT *in vitro* and *in vivo*, indicating that PI3K/Akt signaling could be required by ICT to counteract skeletal muscle atrophy following mechanical unloading.

Skeletal muscles respond to the stimulus of mechanical load for growth and maintenance. Muscle atrophy occurs during the periods of decreased mechanical loading^1^,^2^,^3^,^4^,^5^. The known consequences are loss of muscle mass, decrease in muscle strength and increase in fatigue^6^. Losses of muscle mass and strength decrease the ability to maintain posture and perform a wide range of movements and motions, reduce bone strength^7^ and increase the risk of fractures, incidence of death^8^ and physical frailty^9^. Counteracting muscle atrophy associated with mechanical unloading/inactivity is of great clinical need and challenge. In the past years, physical therapies, such as resistance exercise^10^ and electrical stimulation^11^, are attempted to attenuate muscle atrophy. However, low compliance of patients limits their use of physical therapies. Recently, pharmacological agents, such as insulin-like growth factor-I (IGF-I)^12^, anabolic androgenic steroids^13^, myostatin inhibitors^14^, and β2-adrenergic agonists^15^, have been also tested to counteract muscle atrophy. But, it should be pointed out that potential risks of drug resistance, cardiovascular and prostate cancer and cardiac hypertrophy/dysfunction^16^,^17^, are the bottleneck for their clinical translation. It is desirable to develop an alternative therapeutic agent that could counteract muscle atrophy following mechanical unloading in safety.

Phosphatidylinositol 3 kinase (PI3K)/Akt signaling pathway is critical for regulating the balance between protein synthesis and degradation during disuse/inactivity-induced muscle atrophy^18^. Deactivation of PI3K/Akt pathway not only leads to decreased protein synthesis, but causes increase in protein degradation rate, resulting in muscle atrophy^19^. *Epimedium*, a traditional Chinese herb, has been widely used for both maintaining musculoskeletal function and treating musculoskeletal disorders in safety in China for over 2000 years^20^. Our randomized, double-blind and placebo-controlled clinical trial supervised in late postmenopausal women has shown that oral administration of *Epimedium*-derived flavonoids (EF) for 24 months has no dominant side effects on the major systems, including cardiovascular, reproductive, digestive, and nervous systems^21^, and there is no evidence showing that EF has the potential risk of drug resistance. Icariin is the most abundant active component in *Epimedium*^22^,^23^. Icaritin (ICT), an intestinal metabolite of EF, has demonstrated many pharmacological and biological activities (Fig. 1A)^22^,^23^,^24^,^25^,^26^. It has been reported ICT could cause significant activation of the serine/threonine kinase Akt in human lung epithelial cells^27^. Several studies show that icariin, which has parallel biological activities to ICT, can increase the phosphorylation (activation) of Akt in PC12 (pheochromocytoma) cells^28^, endothelial progenitor cells^29^, rat bone marrow stromal cells^30^, and lipopolysaccharide (LPS)-stimulated macrophages^31^. Administration of LY294002 or wortmannin, specific inhibitors of PI3K, could significantly inhibit the effect of ICT or icariin treatment^27^,^28^,^29^,^30^,^31^, suggesting that PI3K/Akt signaling pathway might be involved in various biological effects of ICT. These clues inspire a hypothesis that ICT could require PI3K/Akt signaling pathway to counteract skeletal muscle atrophy induced by mechanical unloading.

In the present study, we investigated the effect of ICT with or without PI3K inhibitor on protein synthesis/degradation, PI3K/Akt signaling pathway, muscle type distribution, histomorphology and mechanical function in skeletal muscles during mechanical unloading.

## Results

### Effect of ICT on PI3K/Akt signaling in C2C12 cells

**Figure 1:**
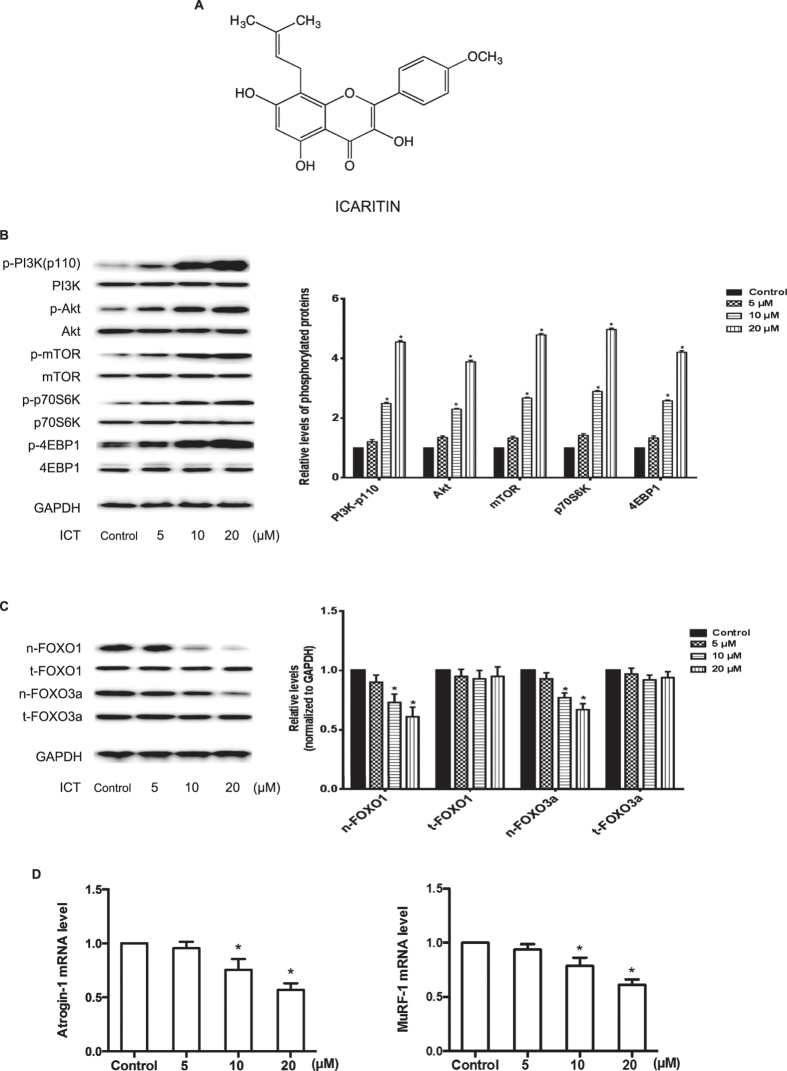
Chemical structure of ICT and effect of ICT on PI3K/Akt signaling in C2C12 cells. **(A)** Chemical structure of ICT. **(B)** Representative Western blots and corresponding densitometry data illustrated the expression levels of phosphorylated and total protein (PI3K-p110, Akt, mTOR, p70S6K, and 4EBP1) in C2C12 cells. **(C)** Representative Western blots and corresponding densitometry data illustrated the nuclear and total cell FOXO1 and FOXO3a protein levels in C2C12 cell lysis. **(D)** Real-time PCR detected the mRNA levels of Atrogin-1 (left) and MuRF-1 (right) in C2C12 cells. Data are presented as mean ± SEM. *for P < 0.05 vs. Control. GAPDH served as the endogenous control. Each sample was assessed in triplicate.

The C2C12 myoblasts were cultured and treated with 5 μM, 10 μM and 20 μM ICT for 24 hours. The phosphorylated and total protein levels of PI3K at p110 catalytic subunit (PI3K-p110) and its downstream proteins, including Akt, the mammalian target of rapamycin kinase (mTOR), ribosomal protein S6 kinase (p70S6K) and eukaryotic translation initiation factor 4E binding protein 1 (4EBP1)^32^, were detected by Western blotting. The distribution of forkhead box O (FOXO) family transcription factors (FOXO1 and FOXO3a) in the nuclear fraction and total cell were also determined by western blotting. The mRNA levels of muscle atrophy F-box protein (Atrogin-1) and muscle RING finger protein 1 (MuRF-1) were examined by real-time PCR. Comparing to the Control Group, the phosphorylation level of PI3K-p110 in the C2C12 cells incubated with 5 μM, 10 μM and 20 μM ICT was 1.2-fold, 2.3-fold and 4.5-fold higher, respectively. Similar patterns were also observed in the phosphorylation levels of the downstream proteins of PI3K (Akt, mTOR, p70S6K and 4EBP1) (Fig. 1B). On the other hand, the expression levels of FOXO1 and FOXO3a in nuclei fraction in the ICT treatment groups (5–20 μM) were 10–35% lower than those in the Control Group in a dose-dependent manner (Fig. 1C). Similar data were also found in the mRNA levels of Atrogin-1 and MuRF-1 (Fig. 1D). In addition, the C2C12 cells were cultured and treated with 20 μM ICT for 1, 3, 5 and 7 days. Cell viability, morphology and myogenic markers (MyoD and myogenin) levels were determined by cell counting kit-8 (CCK-8) assay, cell morphology examination and Western blotting. The differentiation rate of C2C12 cells with ICT treatment was significantly higher than vehicle control (Supplementary Figure 1).

### Involvement of PI3K-p110 catalytic subunit for effect of ICT in C2C12 cells

**Figure 2:**
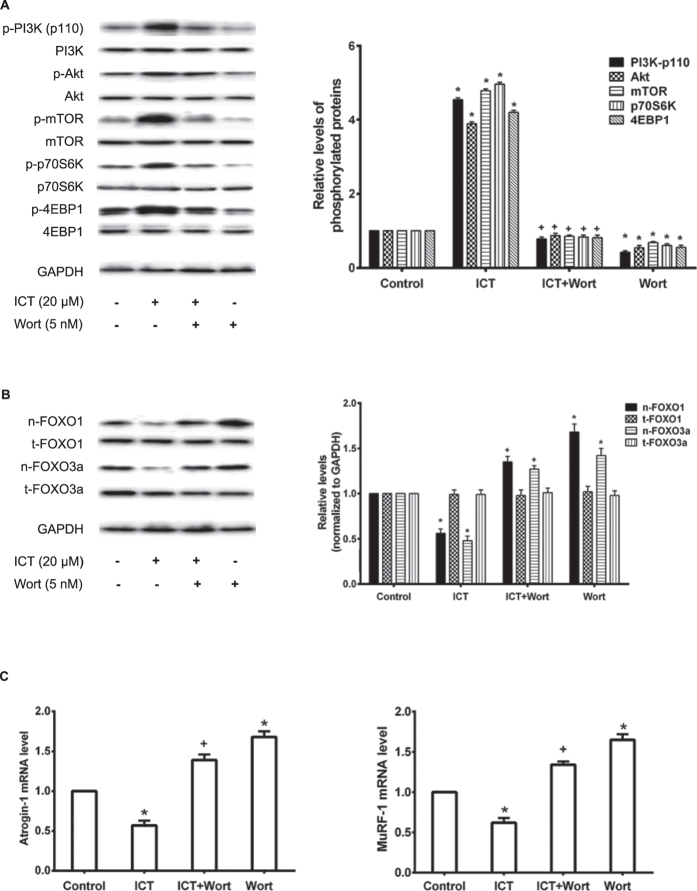
Influence of PI3K inhibitor on ICT effect in C2C12 cells. **(A)** Representative Western blots and corresponding densitometry data illustrated the expression levels of phosphorylated and total protein (PI3K-p110, Akt, mTOR, p70S6K, and 4EBP1) in C2C12 cells. **(B)** Representative Western blots and corresponding densitometry data illustrated the nuclear and total cell FOXO1 and FOXO3a protein levels in C2C12 cell lysis. **(C)** Real-time PCR detected the mRNA levels of Atrogin-1 (left) and MuRF-1 (right) in C2C12 cells. Data are presented as mean ± SEM. *for P < 0.05 vs. Control. + for P < 0.05 vs. ICT. GAPDH served as the endogenous control. Each sample was assessed in triplicate.

To further determine whether PI3K-p110 catalytic subunit is required for the ICT-induced activation of PI3K/Akt signaling in C2C12 cells, a specific inhibitor of PI3K at p110 catalytic subunit wortmannin (Wort, 5nM) was pre-incubated with C2C12 cells for 1 hour followed by the treatment with ICT (20 μM) (ICT+Wort Group) or routine culture medium (Wort Group) for further 24 hours. Cells only treated with ICT (20 μM) or routine culture medium served as the ICT Group or the Control Group, respectively. The phosphorylation levels of PI3K-p110 and its downstream proteins related to protein synthesis in the Wort Group were significantly lower than those in the Control Group. The phosphorylation levels of the proteins in the ICT+Wort Group were significantly lower than those in the ICT Group, but a little higher than those in the Wort Group (*P*  > 0.05). No significant differences were found between the ICT+Wort Group and the Control Group (Fig. 2A). Significantly higher expression levels of FOXO1 and FOXO3a in nuclei fraction were present in the Wort Group compared to the Control Group. The expression levels of FOXO1 and FOXO3a in nuclei fraction were significantly lowered in the ICT Group compared to the Control Group, which was obviously blocked in the ICT+Wort Group (*P* < 0.05). There were no significant differences between the ICT+Wort and the Control Group (Fig. 2B). Similar data were also found in the mRNA levels of Atrogin-1 and MuRF-1 (Fig. 2C).

### Effect of ICT treatment on muscle weight and muscle fiber cross-sectional area (CSA)

**Figure 3:**
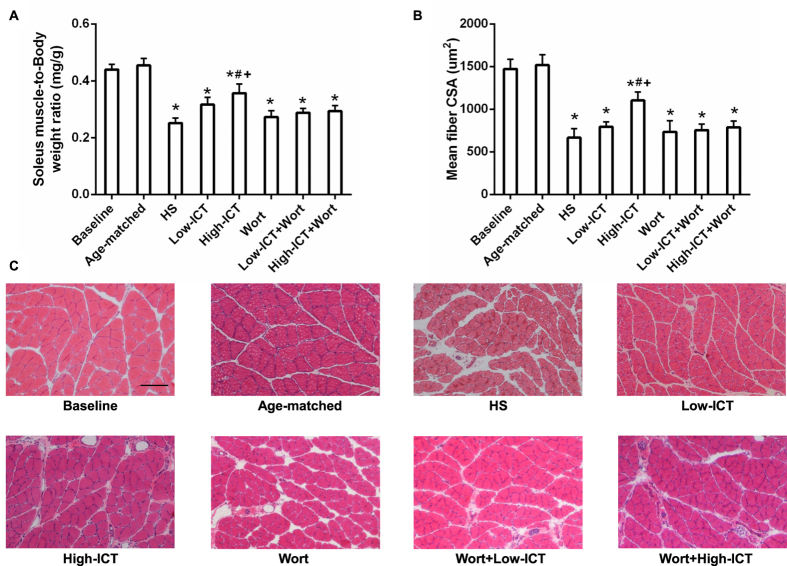
Effect of ICT on muscle mass and muscle fiber CSA. **(A)** Soleus muscle-to-body weight ratio in each group. **(B)** Quantification of the soleus muscle fiber CSA (μM^2^). **(C)** Representative images of cross-sections from mid-belly soleus muscle stained by H&E. Scale bar, 100 μM. Data are presented as mean ± SEM. N = 10. *for P < 0.05 vs. Baseline. ^#^for P < 0.05 vs. HS. + for P < 0.05 vs. High-ICT+Wort. Each sample was assessed in triplicate.

To investigate the effect of ICT treatment on unloading-induced skeletal muscle atrophy, two oral dosages of ICT (80 or 120mg/kg/day) were administered daily to 3-month old male Sprague-Dawley (SD) rats with or without daily injection of wortmannin (15 μg/kg/day) during 28-day hindlimb suspension (HS). The animals were assigned to eight groups including Baseline, Age-mateched Control, HS, Low-ICT, High-ICT, Wort, Low-ICT+Wort, and High-ICT+Wort groups as described in *Materials & Methods*. The muscle-to-body weight (mw/bw) ratio of soleus in the Age-matched Control Group was not changed when compared to the Baseline Group. After 28-day HS, the ratio showed a significant decrease from the baseline in the HS Group. The ratio in the Low-ICT Group was a little higher than that in the HS Group (*P*  > 0.05), whereas the ratio was significantly higher in the High-ICT Group than that in the HS Group. However, the ratio in the High-ICT Group was still significantly lower than that in the Baseline Group. In addition, the ratio in the Wort Group was not significantly different from that in the HS Group. Interestingly, the ratio was significantly lower in the High-ICT+Wort Group than that in the High-ICT Group (Fig. 3A). Similar pattern was also found in the results of muscle fiber CSA (Fig. 3B). There was no significant difference observed in the data of blood biochemical evaluation and histological analysis of the major organs among the HS, Low-ICT and High-ICT Groups (Supplementary Figure 2).

### Effect of ICT treatment on muscle fiber type distribution

**Figure 4:**
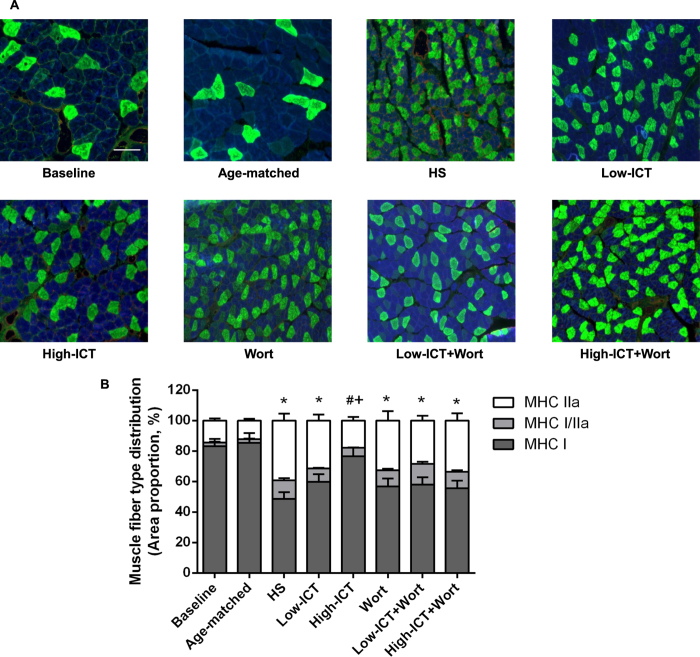
Effect of ICT on muscle fiber type distribution. **(A)** Representative images of cross-sections from mid-belly soleus muscle immunostained for MHC I (blue), MHC IIa (green) and hybrid (MHC I/IIa, light green) muscle fiber. Scale bar, 100 μM. **(B)** Distribution of MHC isoforms in soleus muscle. Data are presented as mean ± SEM. N = 10. Proportion of MHC I fiber, *for P < 0.05 vs. Baseline. ^#^for P < 0.05 vs. HS. + for P < 0.05 vs. High-ICT+Wort. Each sample was assessed in triplicate.

To identify the muscle fiber type distribution, the muscle myosin heavy chain (MHC) type I (slow-twitch type fibers), IIa (fast-twitch type fibers) and IIb (faster-twitch type fibers) were detected by immunohistochemistry staining. As shown in Fig. 4A, the majority of the muscle fiber type in soleus muscle was MHC I fiber. MHC IIb fiber was not detected in soleus muscle while hybrid MHC I/IIa fibers were observed in all groups. There was no significant difference in the proportion of the MHC I fiber between the Baseline Group and the Age-matched Control Group. After 28-day HS, the proportion of the MHC I fiber showed a significant decrease in the HS Group compared to the Baseline Group, while the proportion of the MHC IIa fiber increased instead when compared to the Baseline Group. The proportion of the MHC I fiber in the High-ICT Group was significantly higher than that in the HS Group, which was blocked by the wortmannin treatment (*P* < 0.05). The proportion of MHC I fiber in the Low-ICT Group was moderately higher than that in the HS Group (*P* > 0.05). There were no significant differences observed among three wortmannin-treated groups and the HS Group (Fig. 4B).

### Effect of ICT treatment on muscle function

**Figure 5:**
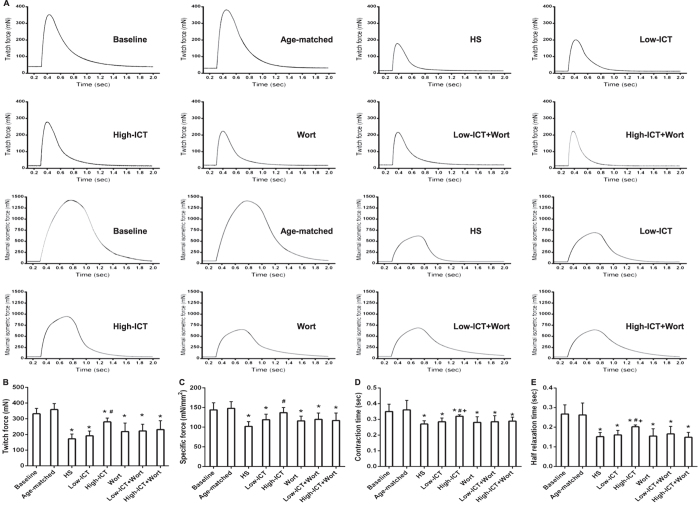
Effect of ICT on muscle function parameters. **(A)** Representative curves of Twitch force (upper two rows) and Maximal isometric force (lower two rows) in each group. **(B)** Twitch force, mN; **(C)** Specific force, mN/mm^2^; **(D)** Twitch contraction time, sec; **(E)** Half relaxation time, sec. Data are presented as mean ± SEM. N = 10. *for P < 0.05 vs. Baseline. ^#^for P < 0.05 vs. HS. + for P < 0.05 vs. High-ICT+Wort. Each sample was assessed in triplicate.

The influence of ICT treatment on muscle functional properties was evaluated with *in vitro* muscle functional testing. The twitch force and specific force in the HS Group were significantly lower than those in the Baseline Group. Both parameters were moderately higher in the Low-ICT Group (*P* > 0.05), but significantly higher in the High-ICT Group compared to the HS Group. The twitch force in the High-ICT was still lower than that in the Baseline Group, whereas the specific force in the High-ICT Group was not significantly different from that in the Baseline Group. The contraction time and half relaxation time were shorter in the HS Group than those in the Baseline Group, while both parameters were prolonged in the High-ICT Group, but still shorter compared to the Baseline Group. Wortmannin treatment led to a moderately lower twitch force and specific force (*P* > 0.05) and significantly shorter contraction time and half relaxation time compared to the High-ICT Group. All the parameters in the three wortmannin treatment groups were not significantly different from those in the HS Group (Fig. 5).

### Effect of ICT treatment on protein synthesis markers

**Figure 6:**
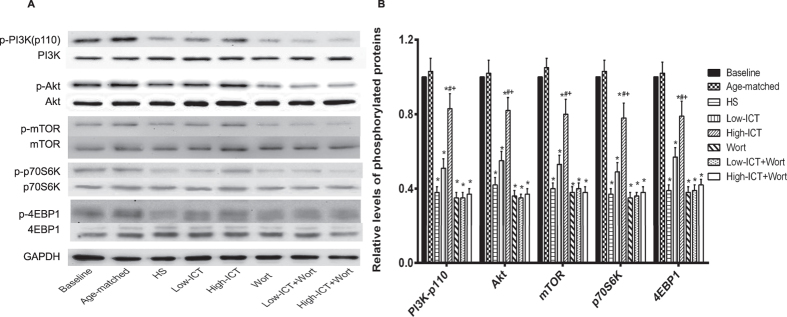
**Effect of ICT on protein synthesis markers**. **(A)** The expression levels of phosphorylated and total proteins (PI3K-p110, Akt, mTOR, p70S6K and 4EBP1) in soleus muscle detected by western blotting. **(B)** Quantification of above marker proteins in soleus muscle. Data are presented as mean ± SEM. N = 10. *for P < 0.05 vs. Baseline. ^#^for P < 0.05 vs. HS. + for P < 0.05 vs. High-ICT+Wort. GAPDH served as the endogenous control. Each sample was assessed in triplicate.

The influence of ICT treatment on the phosphorylation state (activation) of PI3K-110 and its downstream proteins (Akt, mTOR, p70S6K, 4EBP1) that contribute to the protein synthesis in soleus muscle was determined by western blotting. Compared to the Baseline Group, the phosphorylation levels of above markers were significantly lower in the HS Group. The phosphorylation levels of the proteins in the Low-ICT Group were a little higher than those in the HS Group (*P* > 0.05). The High-ICT Group demonstrated significantly higher phosphorylation levels than those in the HS Group, which were significantly attenuated by the wortmannin treatment. The phosphorylation levels of those proteins in the three wortmannin-treated groups were not statistically different from those in the HS Group (Fig. 6).

### Effect of ICT treatment on protein degradation markers

**Figure 7:**
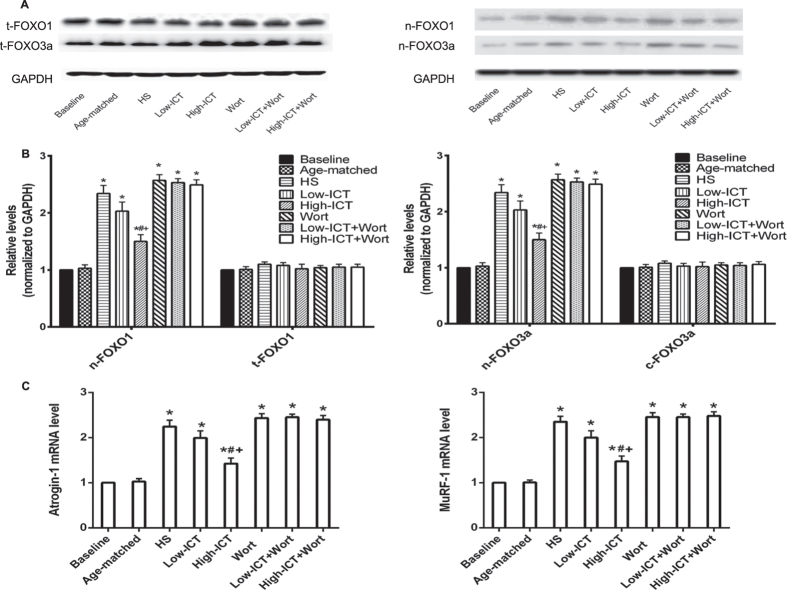
Effect of ICT on protein degradation markers. **(A)** The protein levels of FOXO1 and FOXO3a in total cell (left) and nuclei (right) examined by western blotting. **(B)** Quantification of the proteins levels of FOXO1 (left) and FOXO3a (right). **(C)** The mRNA levels of Atrogin-1 (left) and MuRF-1(right) in soleus muscle detected by real-time PCR. Data are presented as mean ± SEM. N = 10. *for P < 0.05 vs. Baseline. ^#^for P < 0.05 vs. HS. + for P < 0.05 vs. High-ICT+Wort. GAPDH served as the endogenous control. Each sample was assessed in triplicate.

FOXO1 and FOXO3a would be dephosphorylated and enter into the nucleus when Akt was inactivated, then activate atrophy-induced genes, such as muscle-specific E3 ubiquitin ligases Atrogin-1 and MuRF-1 and trigger protein degradation^19^. The cellular distribution of FOXO1/FOXO3a proteins and the mRNA levels of Atrogin-1 and MuRF-1 were detected by western blotting and real-time PCR, respectively. The expression levels of FOXO1 and FOXO3a in nuclei fraction were significantly higher in the HS Group compared to the Baseline Group. The expression levels in the Low-ICT Group were slightly lower than those in the HS Group (*P* >0.05), while the levels in the High-ICT Group were significantly lower than those in the HS Group. Wortmannin treatment led to the significantly higher levels of FOXO1 and FOXO3a in nuclei fraction compared to the High-ICT Group. No differences were found between the Wort Groups and the HS Group. Similar data were also found in the mRNA levels of Atrogin-1 and MuRF-1 (Fig. 7).

## Discussion

To our knowledge, the current study is the first one to demonstrate that ICT could require PI3K/Akt signaling to attenuate the skeletal muscle atrophy induced by mechanical unloading in a dose-dependent manner in adult rats.

After 28-day HS, declines of 42.7% in muscle mass and 54.7% in muscle fiber CSA were observed, which were consistent with the previous findings^11^. An attenuation effect of ICT treatment on muscle atrophy during mechanical unloading was demonstrated. The high dose ICT treated animals showed 25.4% higher in muscle mass and 48.1% higher in muscle fiber CSA compared to the HS animals, the low dose ICT treatment showed moderate attenuation effect. It indicated that ICT could attenuate the decreases of muscle mass and muscle CSA induced by mechanical unloading in a dose-dependent manner.

As an antigravity muscle, soleus muscle consists of mostly type I (slow-twitch) fibers^33^. During unloading-induced muscle atrophy, a slow-to-fast fiber type (type I to II) transition was noticed in our study, which was consistent with the previous reports^5^,^34^. The low dose ICT treatment moderately attenuated the transition compared to the HS Group, whereas the high dose ICT significantly attenuated the transition in the current study. Consistently, the muscle functional analysis showed that the twitch contraction time, half relaxation time, twitch peak force and tetanic peak force decreased during unloading-induced muscle atrophy, which were all attenuated by the ICT treatment. These evidences indicated that ICT treatment could attenuate this slow-to-fast fiber type transition and the decrease of muscle function in a dose-dependent manner.

Inconsistently, the specific force in the High-ICT Group was close to that of the Baseline Group, indicating ICT treatment could prevent the decrease of the specific force during unloading-induced muscle atrophy. The specific force reflects the functional quality of the muscle, maximum specific force in single skeletal muscle fibers is dependent on the number of cross bridges per half sarcomere, the average force per cross bridge, and the fraction of cross bridges in the force generating state^35^. Regarding to the incomplete prevention effect of ICT on the structural features including the muscle mass, muscle fiber CSA and muscle fiber type distribution during mechanical unloading, the inconsistence between the structural and functional consequences might be due to the involvement of more than one mechanism, for example, the regulation of ICT on the excitation-contraction (E-C) coupling properties during unloading-induced muscle atrophy.

Furthermore, the safety evaluation showed no dominant side effects on blood biochemical parameters and major organs during the ICT treatment. ICT could be a potential candidate for counteracting muscle atrophy induced by mechanical unloading.

PI3K/Akt signaling pathway is critical for regulating the balance between protein synthesis and degradation. Activation of PI3K leads to activation of the serine/threonine kinase Akt, which in turn phosphorylates and activates its downstream proteins (mTOR, p70S6K, 4EBP1 and eIF4E) and result in increased protein synthesis^36^,^37^. Our *in vitro* study showed that ICT treatment could promote the expression of PI3K/Akt signaling markers. Consistent with previous reports^38^,^39^, the phosphorylation levels of PI3K at p110 catalytic subunit, Akt and the downstream markers related to protein synthesis decreased after 28-day HS, which could be attenuated by ICT treatment in a dose-dependent manner in our study. On the other hand, PI3K/Akt pathway also could inhibit the process of protein degradation^19^. Deactivation of Akt would lead to relocation of FOXO family transcription factors into the nucleus and activates atrophy-induced genes, such as muscle-specific E3 ubiquitin ligases Atrogin-1/MAFbx and MuRF-1, which contributes to the protein degradation during the progression of muscle atrophy^19^,^40^,^41^. In our study, ICT treatment could attenuate the relocation of FOXO1 and FOXO3a to the nucleus and then depress the elevation of mRNA expression of Atrogin-1 and MuRF-1. Taken together, ICT could counteract the reduction in the activity of PI3K/Akt signaling pathway during unloading.

Wortmannin is a specific PI3K inhibitor, which binds to the p110 catalytic subunit, noncompetitively and irreversibly inhibiting the enzyme^42^,^43^. The expression level of phosphorylated p110 catalytic subunit of PI3K in the ICT+Wort Group *in vitro* (High-ICT+Wort Group *in vivo*) was significantly lower than that in the ICT Group *in vitro* (High-ICT Group *in vivo*). Consistently, the counteraction effect of ICT treatment on unloading-induced muscle atrophy could also be abolished by wortmannin, as evidenced by the lower muscle mass, reduced muscle fiber CSA, more slow-to-fast transition and decreased muscle function in the High-ICT+Wort Group when compared to the High-ICT Group. These evidences indicated that PI3K at p110 catalytic subunit in the PI3K/Akt signaling was required by ICT to attenuate unloading-induced muscle atrophy.

Theoretically, the muscle atrophy should be more serious once the wortmannin treatment and HS were combined. Interestingly, there was no significant difference in all the examined parameters between the Wort Group and the HS Group. PI3K/Akt signaling pathway is crucial for regulating both protein synthesis and degradation during muscle atrophy. Although there are other signaling pathways contributing to muscle atrophy as well^44^,^45^,^46^, PI3K/Akt signaling pathway might play the dominant role during the muscle atrophy induced by mechanical unloading^47^. There’s no significant difference between the activation levels of PI3K in HS group and HS plus wortmannin group, indicating wortmannin treatment plus HS does not have a superimposition effect. It implied that PI3K/Akt signaling pathway inhibition might be mainly required by HS to do harm to muscle.

In summary, our findings demonstrated that ICT treatment had an attenuation effect on mechanical unloading-induced muscle atrophy in a dose-dependent manner and PI3K/Akt signaling was required by ICT to exert its therapeutic effect.

## Materials & Methods

### Cell Culture

C2C12 cells from the American Type Culture Collection (ATCC) were maintained in Dulbecco’s Modified Eagle Medium (DMEM, Invitrogen) which containing 10% FBS (Gibco) and 1% penicillin and streptomycin (Invitrogen) under standard cell culture conditions of 5% CO2 and 95% humidity. ICT (U-sea Biotech, Shanghai, China) was supplemented at concentrations of 5 μM, 10 μM or 20 μM. After 24 hours incubation, cells were collected and lysed for western blotting and real-time PCR analysis. In addition, C2C12 cells were incubated with 20 μM ICT for 1, 3, 5 and 7 days. CCK-8 assay (Sigma-Aldrich, Saint Louis, MO, USA) and morphology examination were performed according to the manufacturer’s instructions. The expression levels of MyoD and myogenin in C2C12 cells were determined by western blotting. In wortmannin experiments, C2C12 cells were pre-incubated with wortmannin (Cayman Chemical, Ann Arbor, MI, USA) at the concentration of 5 nM for 1 hour before the ICT treatment (20 μM). Cells cultured in the routine medium only throughout experiment served as the control.

### Animal model and group designation

Eighty 3-month old male SD rats were recruited and divided into the following eight groups (n = 10 per group): 1) Baseline Group: sacrificed before HS; 2) Age-matched Control Group: weight bearing throughout experiment; 3) HS Group: HS plus vehicles served as treatment control; 4) Low-ICT Group: HS plus ICT at low dose (80mg/kg/day); 5) High-ICT Group: HS plus ICT at high dose (120mg/kg/day); 6) Low-ICT+Wort Group: HS plus Low-ICT plus Wortmannin (15 μg/kg/day); 7) High-ICT+Wort Group: HS plus High-ICT plus Wortmannin; 8) Wort Group: HS plus Wortmannin. ICT was orally administered daily throughout HS. Wortmannin was intraperitoneally injected daily throughout HS^48^. All the procedures used in animal studies were carried out in accordance with the Animal Experimentation Regulations at The Chinese University of Hong Kong and The Hong Kong Code of Practice for Care and Use of Animals for Experimental Purposes. All experimental protocols were reviewed and approved by the Animal Experimentation Ethics Committee of The Chinese University of Hong Kong (Ref No. 12/019/GRF-5). At the end of hindlimb suspension, all the animals were sacrificed with overdose of sodium pentobarbital (35mg/kg) (Sigma-Aldrich, Saint Louis, MO, USA). One side of soleus muscle was subjected to the *in vitro* functional testing and subsequent histology and immunohistochemistry analysis. The other side of soleus muscle was quickly frozen in liquid nitrogen for protein and total RNA extraction followed by the western blotting and real-time PCR analysis, respectively. For safety evaluation, the blood sample was obtained from the HS, Low-ICT and High-ICT groups in order to determine the biochemical parameters. The heart, liver, kidneys, lungs and brain from the above three groups were dissected out, washed with saline and conserved in 10% formalin for histological analysis.

### Hindlimb suspension procedure

The animals were subjected to hindlimb suspension for 28 days following established procedure^11^,^49^. Briefly, a strip of adhesive tape (15 cm × 0.5 cm) was applied to the animal’s tail, which was suspended by passing the tape through a fish-line swivel that was attached to a metal bar on the top of the cage. This allowed the forelimbs to have contact with the grid floor and allowed the animals to move around the cage for free access to food and water. The suspension height was adjusted to prevent the hindlimbs from touching any supporting surface while maintaining a suspension angle of approximately 30°. The animal’s overall appearance, drinking and eating habits and tail were monitored four times per day. The distal tip of the tail was examined to ensure that the procedure did not occlude blood flow to the tail by maintaining the tail pink.

### *In vitro* muscle functional testing

The *in vitro* muscle functional testing was performed following our established protocol^50^,^51^. Dissected muscle with intact tendons was mounted in a specific chamber bathed with 95%O_2_/5%CO_2_-bubbled Krebs’ solution (*In Vitro* Muscle Test System 1205, Aurora Scientific Inc., Aurora, ON, Canada). One end of the tendon was attached to a hook connected to the lever arm of a position feedback motor, and the other end was attached to a force transducer. The muscle was adjusted to the optimum length (L_0_) at which the maximal twitch force was produced. The muscle was stimulated with 0.5 ms pulses of supramaximal intensity through two platinum plates that were parallel to the muscle. Peak twitch force at 1Hz and peak tetanic force at 100Hz for 400 ms were recorded, and twitch contraction time and half relaxation time were calculated with the ASI Dynamic Muscle Control Software (DMC v5.1 beta, Aurora Scientific Inc.). Specific force was expressed as peak tetanic force normalized to the muscle physical cross-sectional area^52^.

### Histology and immunohistochemistry

The dissected soleus muscles were snap frozen in liquid nitrogen-cooled isopentane and then embedded in OCT medium. Serial cross-sections (6 μM thickness) were cut from the mid-belly of the muscles on a cryostat at −20 °C for histological and immunohistochemical staining. Hematoxylin and eosin staining was performed on sections to examine the general morphology and to determine the mean cross-sectional area (CSA) of the muscle fiber.

For immunohistochemical staining, the cryosections were fixed in cold acetone (4 °C) for 10 min and then incubated for 60 min in a 1% bovine serum albumin (BSA)/PBS solution to block non-specific binding. The sections were then incubated overnight at 4 °C with antibody cocktail of primary antibodies against MHC I (BA-F8 1:50, DSHB, University of Iowa, IA, USA), MHC IIa (SC-71 1:600, DSHB), MHC IId/x/IIb (BF-F3 1:100, DSHB). Following three washes in PBS, the sections were incubated with cocktail of Alexa Fluor 350 IgG_2b_, Alexa Fluor 488 IgG_1_ and Alexa Fluor 555 IgM (1:500, Invitrogen) for 60 min. The sections were again washed in PBS, and mounted in Mowiol mounting medium (Merck KGaA, Darmstadt, Germany). Slides were visualized with an Axio Observer Z1 microscope (Carl Zeiss, Jena, Germany) using conventional widefield fluorescence microscopy as well as optical sectioning via structured-illumination fluorescence microscopy (Apotome, Carl Zeiss). Area proportion of each fiber type was determined.

Heart, liver, kidneys, lungs and brain were conserved in 10% formalin for histopathological studies. Tissues were processed by conventional techniques. The paraffine embedded sections of 4–5 μm thickness were prepared with the rotary microtome, stained with hematoxylin and eosin for microscopic examination.

### Western blot analysis

It was to test the activation of PI3K/Akt signaling markers related to protein synthesis and myogenic markers, protein extracted from cell or muscle homogenates was quantified using Bradford Assay (Bio-Rad). Protein samples were separated by SDS-PAGE and transferred into nitrocellulose membranes (Perkin Elmer). After blocking with 5% nonfat dry milk in tris buffered saline (TBS) containing 0.05% Tween 20, the membranes were then probed with primary antibodies against total and phospho-PI3K p110 (Tyr 485) (Santa Cruz), total and phospho-Akt (Ser473), total and phospho-mTOR (Ser2481), total and phospho-p70S6K (Thr389), total and phospho-4EBP1 (Thr37/46) (Cell Signaling Technology, Beverly, MA, USA), MyoD and myogenin (Santa Cruz). Membranes were incubated with specific horseradish peroxidase-conjugated secondary antibodies (Santa Cruz). Immunodetection was performed using enhanced chemiluminescence consistent with the manufacturer’s protocol (BioRad). Membranes were also probed with an antibody for GAPDH (Santa Cruz) to serve as a loading control.

It was to test the cellular distribution of FOXO1 and FOXO3a related to protein degradation, frozen muscle pieces were fractionated into nuclear fraction using a Subcellular Proteo-Extract proteome kit (EBD Biosciences). The proteins extracted from nuclear fraction and total cell were then subjected to the western blot analysis mentioned above.

### Real-time PCR analysis

Total RNA of soleus muscles was extracted using SV total RNA isolation system (Promega), and cDNA was synthesized from 0.5 μg total RNA of each soleus sample using SuperScript III First-Strand Synthesis Kit (Invitrogen) according to manufacturer’s instructions. TaqMan® gene expression assay with primers and MGB probes that are specific for rat Atrogin-1 and MuRF-1 (Assay ID: AF441120 for Atrogin-1 and AY059627 for MuRF-1; Applied Biosystems) were used for real-time PCR analysis. The amplification was performed in a real-time PCR system (StepOnePlus, Applied Biosystems). GAPDH was used as an endogenous control for normalization. Each sample was assessed in triplicate. Relative changes in Atrogin-1 and MuRF-1 gene expression were determined using the 2^−ΔΔCt^ (normalized expression ratio) method of analysis.

### Blood biochemical parameters evaluation

Blood samples without anticoagulant were centrifuged (1000 rpm, 30 min) and the obtained serum was kept at −4 °C for four days, at most, before the biochemical parameters determination. Serum creatinine, urea, uric acid, total protein, alanine aminotransferase (ALT), aspartate aminotransferase (AST) and alkaline phosphatase were determined. Creatinine and total proteins were measured by colorimetric assay, while the other parameters were measured by enzymatic colorimetric assay. The kits used in these tests were obtained from Abcam (UK) and the analysis was carried out using a Multiparametric Autoanalyzer (Lisabio B652, USA).

### Data analysis

The results were presented as mean ± standard error of the means (SEM). The data were analyzed by one way analysis of variance (ANOVA). LSD post-hoc test was made for multiple comparisons among all eight groups to determine the differences at a significance level of P < 0.05.

## Additional Information

**How to cite this article**: ZHANG, Z.-K. *et al.* Icaritin requires Phosphatidylinositol 3 kinase (PI3K)/Akt signaling to counteract skeletal muscle atrophy following mechanical unloading. *Sci. Rep.* **6**, 20300; doi: 10.1038/srep20300 (2016).

## Supplementary Material

Supplementary Information
