# Supplementary material for: Icaritin requires Phosphatidylinositol 3 kinase (PI3K)/Akt signaling to counteract skeletal muscle atrophy following mechanical unloading
Source: Sci Rep. 2016 Feb 2;6:20300. doi: 10.1038/srep20300 (PMC4735824; doi:10.1038/srep20300)
Supplement: Supplementary Information [file srep20300-s1.doc]

**Icaritin requires** **Phosphatidylinositol 3 kinase (PI3K)/Akt signaling to counteract skeletal muscle atrophy following mechanical unloading**

Zong-Kang ZHANG^1,+^, Jie LI^1,+^, Jin LIU^2^, Baosheng GUO^2^, Albert LEUNG^1,*^, Ge ZHANG^2,*^, Bao-Ting ZHANG^1,*^

^1^School of Chinese Medicine, The Chinese University of Hong Kong, Hong Kong SAR; Shenzhen Research Institute, The Chinese University of Hong Kong, Shenzhen, 518057, China

^2^Institute for Advancing Translational Medicine in Bone & Joint Diseases, School of Chinese Medicine, Hong Kong Baptist University, Hong Kong SAR

^+^These authors contributed equally to this work

*Correspondence should be addressed to Bao-Ting ZHANG (zhangbaoting@cuhk.edu.hk), Ge ZHANG (zhangge@hkbu.edu.hk) or Albert LEUNG (awnleung@cuhk.edu.hk).

**Supplementary Figure 1**

**A**

**B**

Day 1 Day 3 Day 5 Day 7

**
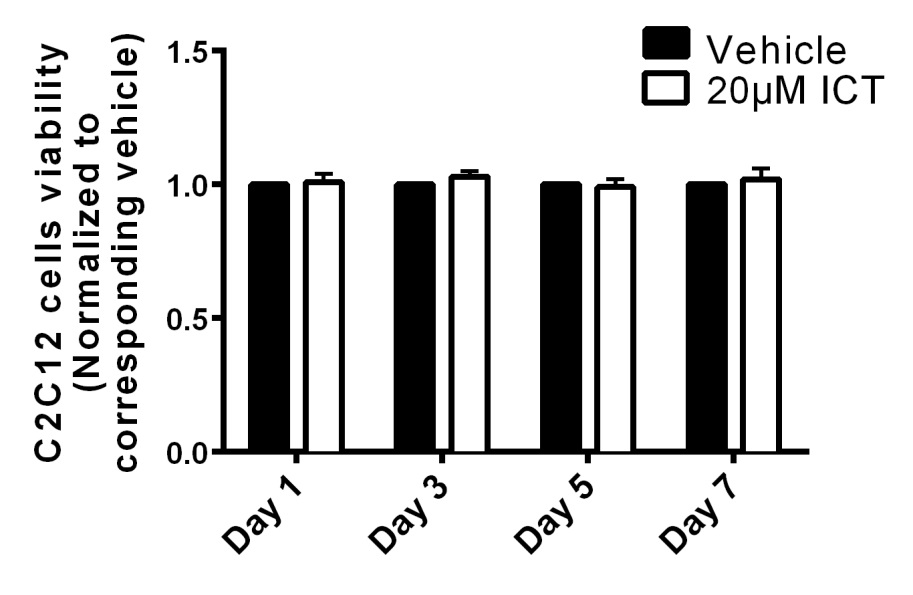
**

**
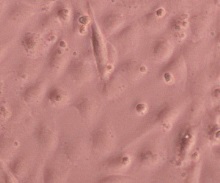

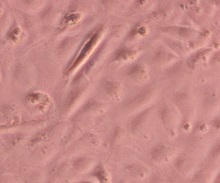

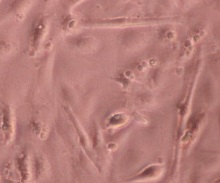

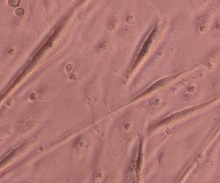
**

Vehicle

20μM ICT

**
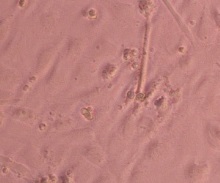

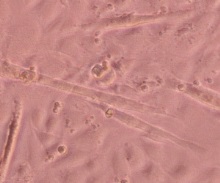

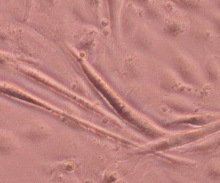

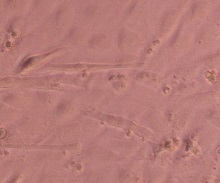
**

ICT

**C**

ICT

ICT

ICT

- **+ - + - + - +**

**
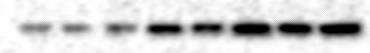
**

MyoD

myogenin

GAPDH

**
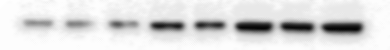
**

**
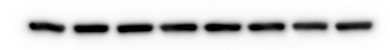
**

Day1 Day3 Day5 Day7

**Supplementary Figure 1 Effect of icaritin on C2C12 cells morphology and differentiation.** (**A**) C2C12 cells viability determined by Cell Counting Kit-8 Assay. (**B**) Representative images of C2C12 cells at 1, 3, 5 and 7 days during differentiation with or without ICT treatment under microscopy. Black arrow: myotube formation. Scale bar, 10 μm. (**C**) Representative Western blots (left) and corresponding densitometry data (middle and right) illustrate the expression levels of MyoD and myogenin in C2C12 cells at 1, 3, 5 and 7 days during differentiation with or without ICT treatment. Data are presented as mean ± SEM. * for P<0.05 vs. corresponding vehicle. # for P<0.05 vs. Day 1 vehicle. + for P<0.05 vs. Day 1 20µM ICT. Each sample was assessed in triplicate.

**Supplementary Figure 2**

**Liver**

**A**

**Heart**

**Kidneys**

**
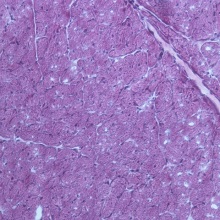

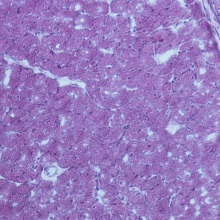

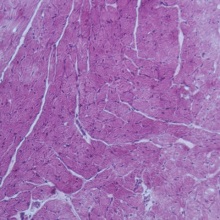

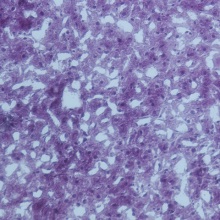

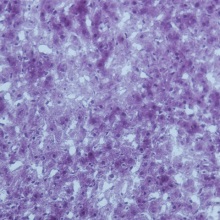

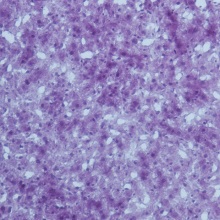

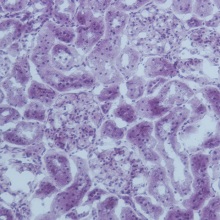

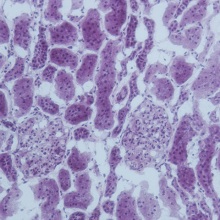

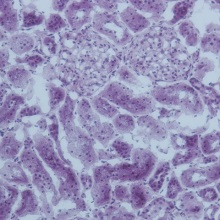
**

HS Low-ICT High-ICT

HS Low-ICT High-ICT

HS Low-ICT High-ICT

**B**

**Lungs**

**Brain**

**
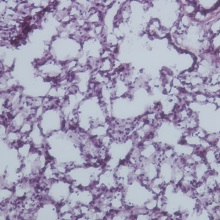

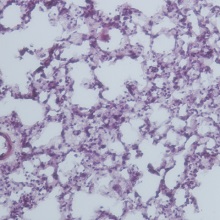

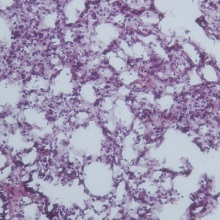

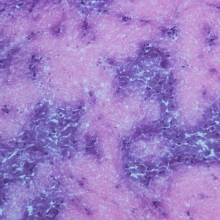

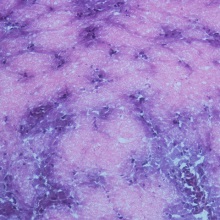

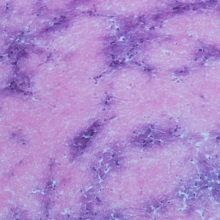
**

HS Low-ICT High-ICT

HS Low-ICT High-ICT

**Supplementary Figure 2 Effect of icaritin on morphology of major organs and levels of blood biochemical parameters.** (**A**) Representative images of sections from heart, liver, kidneys, lungs and brain stained by H&E in each group. Scale bar, 100μm. (**B**) Relative levels of serum creatinine, urea, uric acid, total protein, alanine aminotransferase (ALT), aspartate aminotransferase (AST) and alkaline phosphatase (ALP) from blood samples determined by colormetric assay in each group. Data are presented as mean ± SEM. N=10. No significant differences were found among three groups for the above markers. Each sample was assessed in triplicate.
